# Collaborative and co-Ordinated action for Medication Safety (COMS): Experience-based co-design of an intervention blueprint to improve general practice and community pharmacy collaboration

**DOI:** 10.1371/journal.pone.0338644

**Published:** 2025-12-26

**Authors:** Penny J. Lewis, Christopher J. Armitage, Darren M. Ashcroft, Tom Blakeman, Fay Bradley, Kyle Jarman, Mark Jeffries, Rebecca Morris, Denham Phipps, Andrea D. Short, Steven D. Williams

**Affiliations:** 1 Division of Pharmacy and Optometry, School of Health Sciences, Faculty of Biology, Medicine and Health, University of Manchester, Manchester, United Kingdom; 2 NIHR Greater Manchester Patient Research Collaboration, University of Manchester, Manchester, United Kingdom; 3 Division of Psychology & Mental Health, School of Health Sciences, Faculty of Biology, Medicine and Health, University of Manchester, Manchester, United Kingdom; 4 Division of Population Health, School of Health Sciences, Faculty of Biology, Medicine and Health, University of Manchester, Manchester, United Kingdom; 5 NIHR Applied Research Collaboration - Greater Manchester, University of Manchester, Manchester, United Kingdom; 6 Westbourne Medical Practice, Dorset, Bournemouth, United Kingdom; Jazan Health Cluster, SAUDI ARABIA

## Abstract

Poor communication is a key causal factor of medication safety incidents. Collaboration between community pharmacy (CP) and general practice (GP) staff is essential but hindered by multiple barriers. This study applied an Experience-Based Co-Design (EBCD) approach, incorporating Systems Thinking for Everyday Work (STEW), to develop interventions for improving collaboration and communication on medication safety across the GP-CP interface. A sequential study design was undertaken, including: 1) an experience gathering phase to understand the communication of medication safety issues across the GP-CP interface, involving online focus groups and interviews with 27 GP and CP staff; and 2) two online EBCD workshops with 21 participants, including patients and primary care staff, to generate and prioritise interventions for improving medication safety communication and collaboration. Focus groups, interviews and workshops were audio-recorded, transcribed, and thematically analysed. Three key touchpoints for communication and collaboration on medication safety issues were identified: medication errors, medication changes, and potential patient safety concerns. An absence of shared communication approaches and the prioritisation of medication safety issues, one way communication tools, lack of understanding of professional roles and of incident reporting processes were barriers to communication and collaboration. Facilitators included GP pharmacist-community pharmacist relationships, face-to-face interactions and staff continuity. Five key interventions were suggested: development/modification of an electronic two-way communication tool between GP and CP; centralisation and sharing of patient records; interprofessional education; co-location of general practices and community pharmacies; and a toolkit for improving medication safety across the GP-CP interface. Participants agreed that a toolkit to address key communication and collaboration issues arising at multiple touchpoints should be prioritised for development and discussions led to refinement of ideas and production of a toolkit blueprint. Further research is required to refine toolkit resources, establish an implementation pathway, and evaluate its effectiveness to support adoption and improvements in medication safety.

## Introduction

In England, it is estimated that 237 million medication errors happen each year, and over a third (38.4%) arise in primary care settings. Poor or absent communication is a major contributor to patient safety incidents [1] such as medication errors, particularly across interfaces of care [2]. Problems with communication and collaboration is the factor that patients believe contributes most to medication safety incidents in primary care, including poor or absent communication between healthcare professionals [3].

The World Health Organization highlights the crucial role that collaborative practice and good communication between healthcare professionals has in providing high quality patient care [4]. In recent years, there has been increasing emphasis on improving interprofessional communication, collaboration, and integration within primary care settings [5] – driven by a shift to primary-care delivered healthcare and the introduction of Primary Care Networks across England’s National Health Service (NHS) [6]. Multiple international studies have reported the positive impacts collaborative working between medical doctors and pharmacists can have on patient care [7–9] and subsequently studies have sought to understand the necessary ingredients for their effective collaboration [10–13].

Studies seeking to understand the nature of interactions between general practitioners and community pharmacists in the UK suggest that communication is infrequent, by telephone and confined to routine administrative queries [13, 14]. However, in the UK, the increasing inclusion of pharmacists within general practice (GP) teams has likely altered the dynamic of the GP – community pharmacy (CP) relationship and bridged the gap in communication between these settings [15, 16]. In addition, UK community pharmacies, operating as independent contractors within the National Health Service, provide a range of nationally commissioned clinical services that have broadened the role of pharmacists and driven greater integration with other primary care services. In the wake of the COVID-19 pandemic, communication modes within primary care have adapted, with greater use of digital technologies that can both facilitate and hinder communication and collaboration [17]. These developments may have altered the way in which medication safety incidents and concerns are communicated across the GP-CP interface.

Studies that go beyond simply understanding the nature of the GP-CP relationship to identify how communication and collaboration could be improved provide some useful recommendations for practice [12, 17]. A realist review by Owen-Boukra [12] of GP-CP collaborative and integrated working, made several recommendations for practice including, for example, the need to establish a culture that values and promotes collaborative and integrated ways of working and to establish relationships through repeated interactions [12]. Harris et al [17] undertook a consensus study to develop principles and key actions for collaborative working between English general practices and community pharmacies generating practical actions for implementation [17]. However, despite these insights there is limited understanding of the nature of communication and collaboration regarding medication safety incidents and concerns, and an absence of recommendations that address them.

With its origins in engineering, systems thinking allows for an understanding of complex interactions of multiple elements and individuals. When a systems approach is taken to healthcare service design and delivery it can lead to significant improvements to both patient and service outcomes [18]. Human communication and relationships are the epitome of complexity, making the application of a systems lens to understand this phenomenon especially appropriate. Systems Thinking for Everyday Work (STEW) is a set of principles that provide a practical approach to implementing systems thinking and has been applied to explore system safety in complex environments such as healthcare [19]. A deep understanding of complex systems requires insight into everyday work, and it is through meaningful collaboration with the right stakeholders that we can maximise the potential to develop or identify interventions likely to bring about meaningful change in practice [20]. Consequently, this study applied principles of experience-based co-design (EBCD) along with application of STEW principles, to understand and develop practical interventions to improve collaboration and communication regarding medication safety issues across the GP- CP interface.

## Methods

### Methodological approach

**Fig 1:**
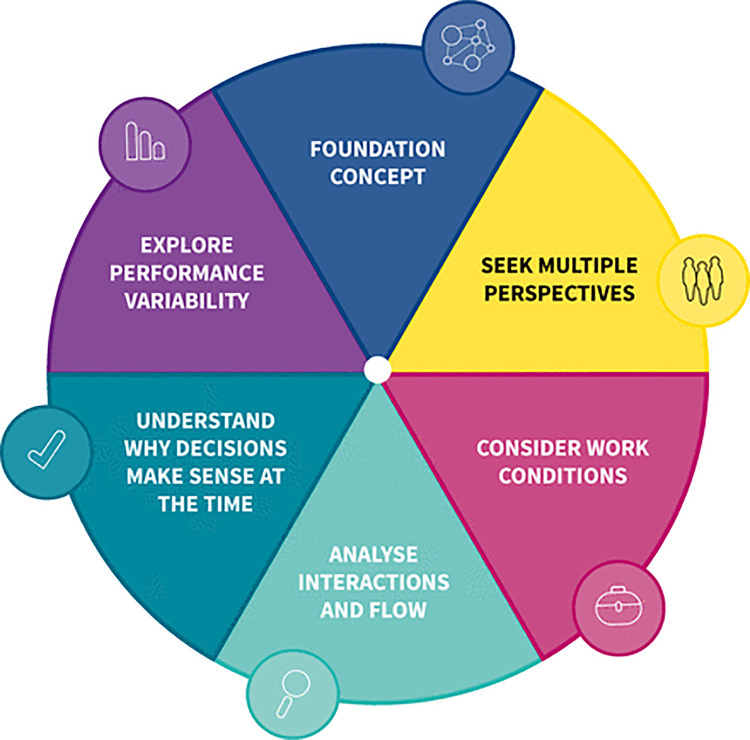
Systems Thinking for Everyday Work (STEW) model.

Experience-based co-design (EBCD) has emerged from the fields of participatory action research and design thinking and is increasingly used to support health system redesign [21] with potential value for intervention design [22]. Experience-based co-design seeks to engage with stakeholders to identify ‘touchpoints’ which are significant points in the interaction between service users and a service or, in this study, between two service providers (GPs and Community Pharmacists). The touchpoints enable the elicitation of areas for improvement. Although EBCD studies are flexible in design [23], there are two key phases: an experience-gathering phase and co-design phase [22]. This sequential study design was undertaken, with an exploratory experience gathering phase followed by two co-design workshops. Ensuring alignment with a systems approach is a key success criterion for co-creation that leads to impactful outcomes [20]. To foster this alignment, Systems Thinking for Everyday Work principles (STEW) principles (Fig 1) underpinned the study design as it is a practical framework that considers the inherent complexity and adaptiveness of settings, such as general practices and community pharmacies [19].

#### Experience gathering.

To explore the nature of communication and collaboration between general practice and community pharmacy in relation to medication safety issues we set out to conduct focus groups, as the group dynamics stimulate discussion, providing an in-depth exploration of multiple perspectives [24].

***Setting, participants and recruitment*:** In line with STEW principles [19], all staff working within the GP or CP setting with a role in medication use were eligible to take part. These included prescription clerks/receptionists, community pharmacists/pharmacy technicians, general practice pharmacists (including prescribers/pharmacy technicians), general practitioners, nursing/allied health professional non-medical prescribers. Recruitment was via the dissemination of a study advert via clinical commissioning networks and existing professional networks in England. Prospective participants received a participant information sheet and those agreeing to take part signed and returned a consent form. Participants received a shopping voucher as a thank you for taking part.

**Fig 2:**
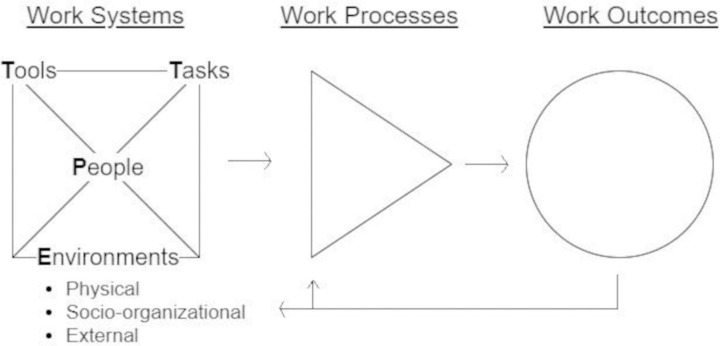
Systems Engineering Initiative for Patient Safety (SEIPS) 101 model.

***Data collection and analysis*:** Systems Engineering Initiative for Patient Safety (SEIPS) 101 model [25] (Fig 2) was selected as a framework for the focus group topic guide as well as guiding data analysis. SEIPs is a widely used framework for unpacking complex socio-technical factors at play in healthcare [25] and complements the principles of STEW [19] with both seeking to understand multiple system perspectives.

At the start of the focus group, participants were asked to collectively generate a list of medication safety issues that they share, or would want to share, across the interface – these were the significant touchpoints of interaction between staff in these settings regarding the management of medication safety issues. Participants were primed to consider their responses to this question prior to attending. Participants were asked to describe the processes involved when managing the identified medication safety issues, the barriers and facilitators and possible improvements. The focus group topic guide can be found in the supplementary material (S1 Fig). Focus groups were facilitated by two members of the research team (PL and FB/MJ) all experienced qualitative researchers and held virtually using the Microsoft Teams platform. Data collection took place from July-September 2022. As focus group attendance proved challenging, we adopted a flexible approach and continued to collect data through dual and one-to-one interviews to ensure inclusion of a breadth of participant perspectives. Transcripts, generated via Teams, were checked and amended through listening to the audio recording before importing into NVivo 12 Plus where they were analysed thematically [26]. This involved an initial reading of four transcripts by PL and KJ, to identify key touchpoints occurring during GPs and CPs work. These were elaborated and grouped as additional data were collected. A coding framework based on SEIPS101 [25] was applied to these four transcripts deductively to identify work system factors at play across these touchpoints. Subthemes within each work system component (i.e., tools, tasks, people, socio-organisational, environment) were then inductively coded by PL, FB and MJ, generating a coding framework that was discussed and agreed with the wider team. This was then applied across all transcripts with an iterative refinement of codes. Data from these thematic analyses were used to inform the co-design workshops, described next.

#### Co-design workshops.

We undertook two co-design workshops to obtain multiple user centred insights and to generate innovative approaches to improvement of communication and collaboration across the GP-CP interface in relation to medicines safety.

***Setting, participants and recruitment*:** Participants were recruited across a range of stakeholder groups with an aim of recruiting 2–3 each of general practitioners, community pharmacists/pharmacy technicians, patients (aged 18 or over) and general practice pharmacists/pharmacy technicians, in addition to 1–2 each of nursing/allied health professional non-medical prescribers and receptionists and a representative from NHS Digital (NHS Digital was the national provider of information, data and IT systems for the NHS. It merged with NHS England on 1st February 2023).

Participants were invited to take part in two, 1.5-hour virtual workshops during November and December 2023 and were recruited through an advert disseminated via social media, professional networks and existing professional contacts of the research team. Following receipt of an expression of interest, prospective participants received a participant information sheet and those agreeing to take part signed and returned a consent form. Participants received a shopping voucher as a thank you for taking part. Participation in both workshops was preferred but was not possible for all participants.

Participants were assigned to workshop breakout rooms based on their stakeholder group, with each room hosting as many different stakeholder roles as possible to ensure a balanced discussion.

***Data collection and analysis*: Workshop 1:** In line with the EBCD approach [23], participants listened to the verbal accounts of healthcare staff (based on excerpts from our prior experience gathering stage) that captured the main medication safety touchpoints across the GP – CP interface (i.e., significant points in the interaction between these settings regarding medication safety). These accounts included what works well and what could be improved, based on specific examples from participants’ experiences. Participants were presented with an overview of the work systems components at play across these touchpoints. Participants worked in breakout rooms to discuss the issues that had been raised, reflect on their own experiences and consider what issues should be prioritised and why. Groups were facilitated by members of the study team (SW, FB, MJ) whose role it was to listen, probe and prompt participants when needed and to provide a verbal synopsis of each group’s discussion with the wider group. Following this, suggested solutions, based on the experience gathering phase, were presented and participants were asked to consider these ideas within facilitated groups along with other possible realistic interventions, tools or solutions to combat the key medication safety issues at these touchpoints. Those with the potential to address multiple touchpoints were particularly sought.

**Workshop 2:** Prior to the second workshop, the solutions and interventions arising from workshop 1 were appraised by the study team according the APEASE criteria (acceptability, practicality, effectiveness, affordability, safety, equity) [27] for their feasibility and potential impact. These interventions were summarised, presented and discussed in workshop 2, with regards to usefulness, practicality and validity, based on the experiences and daily work of the participants. Interventions were prioritised via group discussion among participants at the start of workshop 2 and the key elements of the prioritised intervention were discussed in breakout rooms, again facilitated by members of the study team (PL, FB, MJ). Participants were asked to discuss in groups what the intervention components should look like with a focus on realistic, practical solutions that could be used in the participant’s current work, this led to a blueprint of an intervention. Workshops were audio recorded using MS Teams and transcribed by the research team. Data from workshops were analysed thematically with PL and AS reading transcripts, assigning preliminary codes and grouping into priority areas (workshop 1) and suggestions for improvements and interventions (workshop 1&2). Interpretation of priority areas and solutions was supported by discussions with the wider team (FB, MJ, CA, SW) leading to refinement of the final intervention blueprint. Due to time and resource restraints of both the participants and the project, a celebration event was not held. However, we continue to engage with participants regarding planned follow up work.

As there is currently no published reporting guideline for an experience-based co-design study [22], the consolidated criteria for reporting qualitative research (COREQ) informed the write up of the experience gathering phase [28]. Ethical approval to conduct this study was granted by the University of Manchester Research Ethics Committee: 2022-13935-23438 and 2023-18159-31094.

## Results

### Experience gathering

Data were collected from 27 participants across four focus groups. Due to difficulties arranging suitable times and participant drop out two dual interviews and four one-to-one interviews were also conducted, see Table 1. The vast majority of GP pharmacists and GP pharmacy technicians had experience of working in community pharmacy settings and drew upon their experiences from both settings.

**Table 1:** Focus group/interview participant information.

Focus group/Interview No.	Participant group	Participant code	Sex (M/F)	Years of experience
1	Community pharmacists	CP1	M	20
		CP2	M	10
		CP3	F	15
		CP4	M	20
		CP5	M	3.5
2	Community pharmacists and technicians	CP6	M	8
		CP7	M	13
		CPT1	F	12
		CPT2	F	5
3	GP pharmacy technicians	GPPT3	M	8
		GPPT4	F	3
4	GP pharmacy technicians	GPPT5	F	8
		GPPT6	F	18
5	GP receptionists	R1	F	8
		R2	F	2.5
		R3	F	1
		R4	F	0.5
6	GP receptionist	R5	F	18
7	GP pharmacists	GPP1	M	31
		GPP2	F	20
		GPP3	F	14
		GPP4	F	17
		GPP5	F	10
		GPP6	M	11
8	GP	GP1	M	5
9	GP	GP2	F	10
10	GP	GP3	F	7

Table key: CP = Community Pharmacist,CPT = Community Pharmacy Technician, GPPT = General Practice Pharmacy Technician, R = Receptionist, GPP = General Practice Pharmacist, GP = General Practitioner.

#### Key touch-points.

Communication and collaboration of medication safety issues between community pharmacy staff and general practice staff centred around three issues – these were the key touchpoints of communication and collaboration and were: medication errors (e.g., prescribing, dispensing errors), changes to medication (e.g., on discharge from hospital, after medication review), and potential medication safety concerns (e.g., adherence concerns, stockpiling). The need for change and improvements across all these touchpoints were described by participants.

***Medication errors*:** The communication of actual and potential prescribing errors was the most discussed error, with community pharmacists usually initiating the communication of these. The communication of dispensing errors was also highlighted by both CP and GP staff. Timely resolution, reporting and learning were important to participants, but this was often hindered by poor communication channels and an absence of a shared approach to learning from errors.

***Changes to medication*:** All participants described how changes to patients’ medication made by GP prescribers and after hospital discharge were vital to communicate across the GP-CP interface but that often, this information was not shared in a timely fashion, or at all, leaving patients exposed to medication safety incidents. Furthermore, frequent medication shortages required the temporary substitution of patients’ prescribed medications with suitable alternatives, requiring ongoing co-ordination between prescribers and community pharmacists. This frequent and often disjointed exchange of information and decision-making proved to be a significant source of frustration for both GP and CP participants.

***Potential patient safety concerns*:** CP staff concerns about patients’ safe use of medicines, including suspected non-adherence, were felt by both GP and CP participants to be important issues to communicate across the interface. This included failure to collect prescribed medication or stockpiling of medicines. Whether and how these concerns were shared varied, and there was an absence of systems or processes to facilitate information sharing.

#### Work system components.

Similar system components were at play across all touchpoints with variation in their ability to act as barriers or facilitators to communication dependant on perceived urgency and importance of the medication safety issue at hand. In line with the SEIPs model, these key components (i.e., tools, tasks, people, environment) are described below, illustrative quotations can be found in the supplementary material.

***Tools*:** A range of communication tools were employed across the GP-CP interface, but the telephone was the most frequently utilised medium for communicating medication safety issues. Its use posed significant socio-technical challenges, such as high call volumes, unanswered calls, and delays in response, arising from both technological limitations and organisational factors. The presence of a dedicated telephone line within GP practices was considered helpful, though its use was restricted to the delivery of the community pharmacy consultation service (an advanced pharmacy service offered in primary care in England). Some GP pharmacy teams used WhatsApp, providing a direct channel for CP staff to resolve time pressing medication safety issues, including the handling of medication shortages. However, despite being perceived as an effective means of communication, it was only felt appropriate when staff had a dedicated work telephone.

For medication related issues that were deemed non-urgent, email was often the preferred medium of communication, providing an ‘audit trail’ that both CP staff and GP staff valued. However, several barriers to effective email communication were described, including inconsistent monitoring of emails, a lack of computers, the presence of multiple email addresses for a single CP, and the risk of emails being overlooked during staff absence (when individual email addresses were used). Additionally, concerns were raised regarding the security of email communication, particularly when sent to non-NHS email addresses.

GP pharmacists believed instant messaging was an effective communication tool for addressing medication-related issues within a GP setting and expressed a desire to extend its use to community pharmacies, however, an absence of shared systems within primary care was a recognised barrier to this. As a work around to an absence of shared systems, one GP pharmacy technician described how they had implemented the use of Google© forms to communicate between CP and GP in real-time regarding out-of-stock items.

The introduction of electronic prescribing systems (EPS) was thought to have inadvertently impacted on medication safety communication as it was difficult for prescribers to retract erroneous prescriptions once sent to a CP, in contrast to paper prescriptions that could be physically collected. It meant that it was no longer possible for CP staff to hand back undispensed paper prescriptions as a mechanism of alerting the GP team to possible patient non-adherence.

The one-way functionality of tools, such as electronic prescribing systems, patient summary care records, and community pharmacy consultation service referral systems—prevented two-way communication and reinforced a hierarchical dynamic. Participants believed that, with appropriate governance arrangements, enabling of two-way communication systems and full access to patient records would improve efficiency and medication safety.

An overarching barrier to effective communication was a lack of standardisation in the selection and use of different tools by GP and CP teams leading to staff frustration in both settings.

***Environment: Socio-organisational*:** An implicit hierarchy – with general practitioners seen to possess the most authority and power in relation to medicines use – was perpetuated through system tools and staff attitudes, preventing effective collaboration. Breaking down this hierarchy through greater sharing of information including patient safety incidents, understanding of staff responsibilities and expertise were seen as key to addressing this imbalance and to prevent silo working. However, an understanding and appreciation of pharmacy professionals’ skills had been strengthened with greater inclusion of pharmacy professionals in GP teams.

There was an absence of approaches for sharing analysis and learning from medication safety incidents with some informal sharing described only by those who had established longstanding relationships with their local GP or CP.

A lack of or change in staff and a reliance on locum staff, particularly in community pharmacies, was highlighted as a barrier to collaboration and communication by reducing the opportunities for interaction and inhibiting the development of relationships and trust. A lack of continuity meant that CP staff were unaware of usual and preferred approaches to resolving any given medicines related problem with the general practice.

High workloads and resultant busyness within both settings impacted upon both GP and CP teams’ capacity to communicate effectively. Participants described how the communication of prescription queries, amendments or cancellations was time-consuming due to delayed responses or unanswered phone calls. There was not always time available to discuss long standing, less urgent, medication safety concerns. Community pharmacy opening times was another socio-organisational factor at play, inhibiting community pharmacist attendance at general practice team meetings, reducing opportunities for developing relationships.

Community pharmacists’ ability to make timely clinical decisions was hindered by their limited access to patient clinical records. Ensuring that any out of the ordinary prescriptions were justified by the prescriber on the prescription prevented unnecessary workload for both the community pharmacist- whose responsibility it is to follow up on any prescribing decisions that contravene existing guidelines- and for GP staff.

As previously highlighted, an absence of shared and consistent approaches to communication and the prioritisation of medication safety issues was a significant barrier to effective collaboration. Using an agreed method of communication for a given type of medication safety issue was considered an important approach to improving communication.

***Environment: Physical and external*:** Geographical distance between the CP and GP was a key factor influencing the development of relationships – the communication of medication safety issues was believed to be most effective when community pharmacies were co-located within general practice. Independent community pharmacies were perceived as more proactive in their communication, possibly due to having better staffing levels and greater flexibility in adapting their processes.

National medication shortages were a common external driver for communication across the GP -CP interface requiring effective and frequent communication between settings as poor handling of this issue could lead to patients not receiving necessary medication or receiving duplicate therapy. There was a desire for an efficient way for managing this issue to reduce workload and prevent patient harm.

***People*:** Familiarity was important to effective communication, building trust and a shared understanding that CP and GP teams were working together for patient benefit. Familiarity was engendered via multiple mechanisms, the most prominent being in-person contact and appeared especially important to GP participants; however, this face-to-face contact was not always possible due to distance and/or workload. This familiarity facilitated the communication of safety issues, particularly prescribing errors, by disarming any potential defensive response from GP staff. Clear introductions and cross organisational inductions were facilitators to fostering these relationships and understanding roles.

With their access to patient records, availability, and ‘insider knowledge’ of CP processes, GP pharmacy teams were described as key facilitators to communication and collaboration, acting as boundary spanners to address medication safety issues across the interface. However, there were concerns raised by CP participants that only contacting GP pharmacy teams when errors had arisen, might put this relationship under strain.

Receptionists were perceived by CP staff as gatekeepers that could inhibit effective communication by obstructing or unintentionally misinterpreting messages. Their limited knowledge of medicines was identified as a challenge, and receptionist participants expressed a desire for greater understanding of medication-related processes to facilitate communication. The potential for misinterpretation was also a concern for participants who described how patients were required to act as a ‘go-between’ passing messages between CP staff and GP staff when medication safety issues had been spotted. One GP participant felt there had been an increase since the COVID pandemic in patients relaying messages rather than the pharmacy contacting the general practice directly.

General practitioners’ individual approach to prescription writing, in terms of information provision and clarity varied, but when this information anticipated any potential safety concerns a pharmacist might have, this reduced the need for clarification, saving time for staff and ensuring patients timely access to their medicines.

***Tasks*:** Most tasks described by participants (e.g., prescribing, dispensing) were well structured and frequently carried out with few problems. However, when a problem did arise, a disconnect in workflows of general practice and community pharmacies resulted in a misalignment of communication across the interface causing delays to timely resolution. The differing work systems and a lack of shared socio-organisational processes compounded this issue as well as the high workloads experienced in both settings. Tasks such as prescribing and clinical checking were easier when the CP was co-located in the GP, as described previously.

### Co-design workshops

A total of 21 participants attended workshop one and 18 attended workshop two, with 12 participants attending both workshops (see Table 2). Participants were from nine different counties in England with the highest number from Greater Manchester (n = 8). Target recruitment was reached for all stakeholder groups, with the exception of non-medical nursing or allied health professional prescribers, where recruitment was not successful for either workshop. However, none of the discussions relating to the sharing of medication safety issues involved non-medical prescribers (other than pharmacists who were included in the sample).

**Table 2:** Target and actual number of participants from each stakeholder group at each workshop, including the number of participants that attended both workshops.

	Target	Workshop 1	Workshop 2	Attended both workshops
**General practitioners (GP)**	2-3	3	2	1
**Community pharmacists (CP)**	2-3	2	1	1
**Community pharmacy technicians (CPT)**	2-3	2	2	2
**Patient representative (P)**	2-3	3	4	3
**General practice pharmacists (GPP)**	2-3	3	4	0
**General practice pharmacy technicians (GPPT)**	2-3	3	1	1
**Non-medical prescribers (nursing/allied health professionals)**	1-2	0	0	0
**Receptionists (R)**	1-2	2	2	2
**NHS Digital**	1	1	0	0
**Other**	NA	2*	2*	2
**Total**	14-23	21	18	12

*1 Primary Care Network Pharmacist & programme manager for medicines optimisation, 1 pre-registration pharmacist.

#### Workshop 1 (WS1).

During WS1, four priority areas for intervention were identified by stakeholders. These were: understanding roles and building relationships, communication tools, lack of patient/medication information to make decisions on safe/prompt supply of medicines and shared learning on medication safety issues and their prevention. Illustrative quotes are provided in Table 3.

**Table 3:** Priority areas for intervention and illustrative quotations.

Priority area	Illustrative quotes
Understanding roles and building relationships	*“…if there’s respect and trust between two professionals, regardless of the different roles, then that’s going to enable better working practice. So how do you develop respect and trust? By understanding each other’s working practices and knowing each other in a human way…”* Patient representative
Communication tools	*“I think it [two-way electronic communication] would solve a lot of problems in terms of actually a) people struggling to ring the practice but b) actually having a clinical pharmacist explanation for maybe some prescriptions that may have been bounced back and to know exactly what the issue is. Because sometimes it’s a case of, oh, we don’t have this and they will have suggested alternatives, but it’s gone through reception, the receptionist has spelt it wrong and I have to pick up the phone again to ask what the suggestions were in the first place. So if there was some way of enabling that two-way communication, it would be great.”* General practitioner
Patient/medication information to make decisions on the safe/prompt supply of medicines	*“I will always add more detail [to the prescription] and so say it’s a child, child’s weight, if the kid’s medicine would need to be double checked with the weights, I will add weight onto the prescription into the comment box, you know, to make sure there’s no [prescription] tennis or coming back …but working with other clinicians, they don’t see the work that goes on, what we’ve got to do [in community pharmacy] and phone calls, we’ve got to make to question those prescriptions and because we don’t have the full picture, we don’t have [the] patient record.”* GP practice pharmacist & prescriber
Shared learning regarding medication safety issues and their prevention	*“I don’t think in GP practice we share our errors to community pharmacy, so they’re aware, and you know, maybe from their perspective, we kind of maybe see ourselves as a bit, you know, not to blame for anything…. And I think if we shared more of the errors that have been made, maybe the community pharmacy would feel more comfortable sharing their errors... And I think if you opened that communication between general practice and community pharmacy, it would really be beneficial both ways.”* GP pharmacist

Solutions and facilitators to communication and collaboration of medication safety issues discussed by focus group/interview participants and workshop stakeholders were refined and prioritised within workshop 1. This resulted in five key solutions to improving collaboration and communication:

1. Interprofessional learning resource/toolkit for improving medication safety across the GP-CP interface.
2. Development/modification of an electronic tool for ensuring two-way communication between GP and CP.
3. Centralisation and sharing of patient records.
4. Interprofessional education to facilitate early experience of collaboration.
5. Co-location of general practices and community pharmacies.

These interventions were assessed by the study team according to the APEASE criteria [27]. Based on this process, it was determined that a learning resource/toolkit to address the key communication and collaboration issues arising at multiple touchpoints, should be prioritised for development.

#### Workshop 2 (WS2).

During workshop 2, participants unanimously agreed in the potential benefit of a toolkit and how it could establish ‘*some common ground for doing things*’. However, key recommendations were that toolkit components should be adaptable to local contexts (varying skill mix, resources etc.), resources should be ‘*short and snappy’*, staff of all groups should be involved, and that patient experience should be at the centre of its development. Discussions within breakout rooms led to refinement of ideas for toolkit resources based on key themes generated from the first workshop leading to production of a blueprint of toolkit components (see Table 4).

**Table 4:** Collaborative and co-Ordinated action for Medication Safety (COMS) toolkit blueprint.

Communication Theme	Objective	Toolkit resources	Example quote
**Setting the scene- placing patient care at the centre**	To understand the systems and processes for safe medicines use across both GP and CP through the eyes of patients.	- The patient journey [29]: videos of patients’ experiences navigating the differing and confusing systems and processes of both GP and CP in relation to medicines use. - Key information to improve the patient journey based on common scenarios (e.g., emergency supplies, timely supply of new/amended medicines, opening times) - How to work with patients to understand the specific issues they face within the local context.	*“I love the idea of a patient journey actually having points on the timeline where you can draw up examples and you can go to from the toolkit to where you just need to and you can have different examples of how community pharmacy patient and GP relationships either need to be improved or can be improved and possibly having a few videos and scenarios”* General practitioner *“It’s looking at the consequences and showcasing the consequences of getting things wrong and pointing out where things and disastrous things have happened”* Patient representative *“So if we get them [patient participation group] involved as well from their aspect from patients view, like what’s important to them, what they think is dangerous, What’s concerning them and bring them into the equation as well, that may be good idea.”* General practice pharmacist
**Understanding roles and building relationships**	To build trust and relationships and seek a mutual understanding and clarity of roles.	- Importance of introductions [30], face to face interaction and how to sustain regular contact [31] (e.g., invitations to meetings [12], sharing contact details, changes to staff) - Staff observing practice [15] within the GP/CP. Template of key medication processes to observe and inclusion of period of observation within induction programmes - Video of a typical day in a pharmacy/general practice including key challenges (including patient perspectives) - Template of who to contact regarding specific medication related issues depending on urgency	*“I think it’s really helpful to kind of meet people face to face or at least online, and so maybe that as a suggestion in the toolkit might be good because once you - I’m a GP, so and I’ve met and know one of my local pharmacists really well and I tend to recommend patients looking for a new pharmacy to go to her because I’ve met her because, we’ve discussed things. So just having that kind of connection I think can be really, really helpful.”* General practitioner *“Templates and things that you come up with in the resource packs…a contact for each pharmacy, say, to sort of say to the surgery, right, if you’ve got prescription issues, which email do we use?”* Primary care network pharmacist *“Clear guidance on how each and every Pharmacy operates so the doctors know where they stand, and patients know where they stand.”* Patient representative
**Communication tools and processes**	To generate a joined-up approach to communicating medication safety issues across the GP-CP interface	- Dedicated phone line and/or email to GP/GP pharmacy team/CP to deal with medication safety issues [15] - List of possible communication tools, advantages and disadvantages and points to consider - Template setting out mutual agreement regarding appropriate form of communication for each type of medication issue depending on urgency and importance [31] - Dedicated role with responsibility for contact with CP/GP so they are immediately available (often GP pharmacists but not always) [31] - Standardised template for communicating medication queries regarding specific patients.	*“It’s frustrating not to have that direct channel of communication and it has got better with pharmacy staff and GP’s, but there is still a lot of inconsistency both from pharmacies and from general practice, and I think that that is what we need to sort out is who are the people that if this happens, who do we speak to? if it’s out of stock, what do you want me to do? Do you want me to email it? and that might be different for every practice, but it’s important that we at least establish that.”* Community pharmacist *“If there’s a standardised kind of form it, like a template… that could be: this patient, this is the issue, and just have those set kind of sections in the template then that would go through and, they have to be manned every day so it could then be up to the practice to cascade that to the relevant PCN, pharmacist or practice pharmacist technician...so that would be the easiest way of electronically sending something straight over.”* Community pharmacist
**The importance of patient/medication information to make decisions on safe/prompt supply of medicines**	To promote the sharing of key medicines related information	Guidance on the inclusion of useful information on the prescription, e.g., weight, explanation of unlicensed doses, non- BNF doses (formulary choices), clarity of dosage changes -what should be stopped and started, end dates - Examples of usefulness and importance of prescribing information -Incorporation of prompts for information on electronic prescribing systems - Preventing prescription tennis, useful information for GPs on stock checking, possible solutions such as spreadsheets etc. - Agreed processes for uncollected medicines, handling prescription queries	*“In the toolkit, you can write, what I recommend people to do - write out when this is start, when it’s supposed to stop, when the doses supposed to titrate, or when the monitoring needs to be done, and so if we can put it on the prescription as well... Like today…somebody put on the note saying that it has to be stopped after end of November, pharmacy rung me to say, is it supposed to stopped... So it’s just because someone put a note on there that can be stopped. So if we have something to put down, let’s say start date and the change of dose when or as per what letter, even the system, and people can put it in and they can be printed on a prescription that will reduce the amount of errors.”* General practice pharmacist
**Need for shared learning on medication safety issues and their prevention**	To ensure safety lessons are shared across the whole system	-Information on benefits of sharing learning. - Suggested activity – to share error reporting processes, agreement of sharing processes going forward.	*“I think the lowest hanging fruit that’s tangible for me is sharing sort of critical errors and patient safety errors and even that doesn’t really happen in my practice at the moment. We probably have reasonable relationships with the 10 most pharmacies that I EPS to, but even for very major incidents, they aren’t shared.”* General practitioner *“I suppose a bit of a culture change in terms of communication between general practise and primary care in that for medication and medication errors, for example that we are open and transparent and communicating and part of that is developing that relationship, isn’t it?”* General practice pharmacy technician

**Guidance for toolkit use**: it is a requirement for an initial meeting (this can be online if restrained by geographical constraints although face to face is preferable) between general practice, community pharmacy staff and patient representatives to discuss overall contents of the toolkit and determine which components would be most useful and determine a plan for completion of selected elements.

## Discussion

Interprofessional collaboration in healthcare has been extensively explored and conceptualised through various theoretical lenses, however, to our knowledge, this is the first study to focus exclusively on collaboration and communication between general practice and community pharmacy settings regarding medication safety and to use an EBCD approach to initiate the development of targeted interventions for improvement.

The first phase of the study explored the main medication safety communication touchpoints and elucidated the multiple barriers and facilitators stemming from work system components that staff from both GP and CP settings experienced in their day to practice. The findings corroborated Bradley et al.’s conceptual model of GP-CP collaboration, identifying comparable facilitators such as consistent reciprocal communication, interprofessional familiarity, and trust [32].

The addition of pharmacists within NHS general practice teams had clear positive implications for medication safety communication and, like previous studies, GP pharmacists were thought to provide a better link between GP and CPs [16], particularly as they ‘speak the same language’ [15]. Failure to inform pharmacies of changes to patient medication is a common medication safety issue [32] and was a frequent reason for communication, and like previous research [15], queries about such issues were often handled via contact with the GP pharmacist.

The high workloads of community pharmacies was described by all participant groups as a barrier to effective communication of medication safety issues and is a widely reported global concern [33–35] underpinning safety incidents [36]. These problems are compounded by medication shortages [34], a common reason for communication. The need for interventions that support communication and collaborative processes without adding to existing workload is therefore paramount and, if successful, have the potential to reduce workload by optimising communication processes and reducing failure demand (the demand caused by a failure to do something or do something right so that further demands are made unnecessarily consuming the organisation’s resources [37]).

Participants during the co-design phase of this study, identified five key solutions to improving communication and collaboration, all of which have been advocated by previous studies. One recommendation generated was the development/modification of an electronic tool for ensuring two-way communication between GP and CP and another, was centralisation and sharing of patient records. A lack of integration of communication strategies has been cited as a barrier to information sharing in recent realist review of CP and GP integration [12] and a shared information system has been suggested as an approach to promoting better integration of CP into the primary care pathway [33]. However, careful consideration would need to be given to patient confidentiality and governance [12]. Greater pharmacist access to patient records has also been highlighted by previous research as a facilitator to collaborative patient care [33] and although the introduction of summary care records in the UK has provided pharmacists with some access to patient information, there is clearly a wish for access to more comprehensive information to improve the safe use of medicines. As a work around, the GP pharmacist, being more accessible to CPs, acted as an intermediatory information broker, accessing patient records from the GP surgery when requested to by CPs [15].

The NHS Long Term Plan recognised the importance of interoperability of digital technologies to free up time and resources [6] and, in the recent version of the NHS GP contract, practices will be required to ensure they allow read only access to patients’ care records and for community pharmacy professionals to send consultation summaries to GPs [38]. Although this improved information sharing will be welcome, there needs to be synchronicity of communication systems that support bi-directional information flows between community pharmacies and general practices to lead to meaningful improvements in communication and collaboration across the interface- a call that is echoed internationally [39].

Interprofessional education (IPE) is a mandatory component of undergraduate pharmacy and medicine courses in the UK and is common practice worldwide. It has been reported to improve understanding of professional roles and the skills required for prescribing [40]. Most IPE is undertaken within a simulated university environment, yet IPE delivered within a real-life context of practice might provide better understanding of the systems and context in which collaboration and communication occurs and is an approach that is preferable to students [41]. Greater opportunities for IPE within a clinical environment, including within primary care settings may enhance understanding of safe medication processes and the roles of health care professionals. Although this option was not prioritised for development, the toolkit blueprint includes approaches to improving understanding of GP and CP team roles and processes and importantly includes those staff groups who would not typically undertake IPE, such as receptionists and prescription clerks.

The co-location or close geographical proximity of general practices and community pharmacies is commonly cited as a facilitator for collaboration [31–33] by promoting regular communication, trust [12], and improved workflow. In particular, GP participants explained how they valued regular face to face contact with CP staff, a finding highlighted by Hindi et al. [33] The mass relocation of CPs and GPs is clearly impractical; however this study echoes the sentiments of those before it, that future building and renovation plans should consider co-location of premises.

After applying the APEASE criteria, the solution selected for further consideration was an interprofessional toolkit for improving medication safety across the GP-CP interface. This toolkit sought to cover multiple touchpoints and provide a practical approach to improving safe medicines use in the short and long term. The development of a toolkit is an approach particularly useful to tackle complex issues where there are inherent variations in context and processes, for example, the repeat prescribing toolkit published by the Royal Pharmaceutical Society and the Royal College of General Practitioners provides general practices and community pharmacies the first framework for improving the efficiency and safety of repeat prescribing [42].

The COMs toolkit blueprint seeks to facilitate communication and collaboration in relation to medication safety by developing and strengthening communication approaches and promoting a culture of collaboration which may, in turn, pave the way for future wider integration [12]. This will be achieved with both GP and CP teams working towards the shared goal of promoting patient safety: a fundamental principle of medicine, is ‘do no harm’ and, with the focus on this central tenant of medical practice, it is hoped inclination to defend professional territories will be overridden. The need for a shared ambition is a key dimension of collaboration in healthcare [43], therefore a resource that is useful to both GP and CP teams in improving effectiveness of medication related processes and that seeks to improve patient safety will hopefully be perceived as ‘useful’- an important driver for in collaboration [31]. Participants emphasised the importance of future toolkit users understanding the patient’s perspective of medication use, a valuable approach for examining collaboration and communication among health professionals and highlighting possible gaps in care [44]. It would also reinforce the importance of patient-centred decision-making and reduce silo working.

Learning from medication safety incidents was highlighted as area where greater communication and collaboration should be developed. As found in this study, communication and learning from incidents has been found to arise in professional silos [45]. Participants wanted to understand more about the safety management processes used by local GPs or CPs but more importantly felt that this might be an opportunity to share good practice and make recommendations for safety that could have implications for both organisations. Communication across the GP-CP interface typically only arose in reaction to actual or potential safety risks – backing the notion that communication is perceived as not necessary when things are going well [13, 32]. However, learning from how things go well, particularly in challenging and frequently changing situations [46] can lead to system resilience [47] and can be engendered by communication and collaboration that seeks to anticipate challenges and to adapt accordingly. One example in this study was the use of a shared document set up by CPs and used by GP staff that indicated current medication stock, preventing ‘prescription tennis’ and saving time.

The resulting toolkit blueprint sets out a framework for a suite of resources that can be tailored to the individual context of a general practice and community pharmacy. It is not intended that the toolkit provides a prescriptive, one size fits all set of resources, and such rigid and untailored interventions are not recommended [48]. What our toolkit seeks to foster is a shared understanding of the medication related systems and processes present across these key primary care organisations and where there are no systems, a shared agenda for ensuring safe processes and care. Future work needs to develop and refine the toolkit resources and a pathway to implementation and evaluation to promote national uptake. An important factor to consider in the toolkit design is its ability to be resilient to changing demand and resources- therefore, close monitoring and review of its implementation would be required.

### Strengths and limitations

Utilisation of an EBCD approach and systems thinking meant that our understanding of this phenomenon was grounded in the reality of everyday practice and incorporated multiple perspectives of those involved in the safe use of medication in general practice and community pharmacy. The use of the SEIPs101 model facilitated a breakdown of key components within the complex systems of GP and CP and enabled visualisation of common factors that hinder and facilitate communication. This led to a robust toolkit blueprint that has a system-wide and practical approach to improving communication and collaboration across the GP-CP interface. Although the toolkit blueprint was developed based on the perspectives of patients and professionals in England, the drivers for each toolkit domain resonate with issues experienced within primary care internationally. However, the study had several limitations which may limit generalisability, including a small sample size considering the heterogeneity of roles and contexts and the possibility of self-selection and social desirability bias. Furthermore, due to difficulties in scheduling busy healthcare professionals, we had to take a pragmatic approach to data collection, carrying out interviews rather than focus groups on some occasions. Although the interviews provided useful insights, they relied on greater probing by the interviewer resulting in less richness in the data.

## Conclusion

This study has led to the development of a blueprint for a toolkit that aims to improve collaboration and communication of medication safety issues across the general practice interface. Our EBCD approach, involving a diverse range of participants, supported the development of toolkit components that not only tackle pertinent medication safety issues but are acceptable to stakeholders and thus more likely to be implemented in healthcare.

## Supporting information

S1 Fig Focus group topic guide. (DOCX)

S1 Table Work system components- illustrative quotes. (DOCX)
