# Supplementary material for: Collaborative and co-Ordinated action for Medication Safety (COMS): Experience-based co-design of an intervention blueprint to improve general practice and community pharmacy collaboration
Source: PLoS One. 2025 Dec 26;20(12):e0338644. doi: 10.1371/journal.pone.0338644 (PMC12742773; doi:10.1371/journal.pone.0338644)
Supplement: S1 Fig — (DOCX) [file pone.0338644.s001.docx]

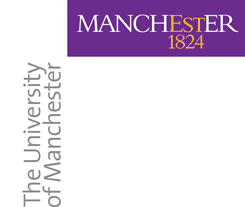


**Focus Group Topic Guide**

Welcome and thank you for volunteering to take part in this focus group, I know you are all very busy. You have been asked to participate as your views on the topic of communication will be very valuable to this research and will help us in our ambitions to produce a set of interventions to improve communication across the GP-CP interface.

**Anonymity:** Despite being recorded, I would like to assure you that the discussion will be kept confidential. The audio files and associated transcripts will be stored securely on a password protected computer, then after the data have been analysed and published, the audio files will be destroyed. The transcribed notes of the focus group will contain no information that would allow named people to be linked to specific statements. You should try to answer and comment as accurately and truthfully as possible. I and the other focus group participants would appreciate it if you would refrain from discussing the comments of other group members outside the focus group. If there are any questions or discussions that you do not wish to answer or participate in, you do not have to do so; however please try to answer and be as involved and inclusive as possible.

**Ground rules**

- The most important rule is that only one person speaks at a time. There may be a temptation to jump in when someone is talking but please wait until they have finished.
- There is no right or wrong answer.
- You do not have to speak in any particular order.
- When you do have something to say, please do so. There are many of you in the group and it is important that I obtain the views of each of you.
- You do not have to agree with the views of other people in the group.
- Does anyone have any questions? (answers).
- OK, let’s begin.

**Is it ok to start recording?**

**[Start by everyone introducing themselves, area of work, previous experience and how long been a pharmacist?]**

**Introductory question**

To start, we are going to produce a list of medication safety issues that you currently share or would like to share across the GP-CP interface [go around the group in turn]

- Could you describe any times where information has not been shared across the GP-CP interface that has led to repeated safety errors?

**Make a list of suggested safety issues.**

**Take each safety issue one by one through the following questioning:**

1. What are the outcomes you seek to achieve from sharing this type of information? [Outcome]

2. Are these outcomes shared?

3. What are the processes involved in sharing/attempting to share this type of information across the GP-CP interface?

4. Explore the work system – i.e. people, environment, tools, tasks for each

5. Ask participants to reflect on examples of when communication of [safety issue] goes well and when it doesn’t

6. What are the barriers to communication of [medication safety issue] across the GP-CP interface?

7. What are the facilitators to communication of [above medication safety issue] across the GP-CP interface?

8. Possible impact of Covid on the work system (work variability)

9. How NHS England/ Digital could improve interoperability of computerised systems between GP and Pharmacy silos at primary care level to improve communications and patient data recording?

**Concluding question**

Of all the things we’ve discussed today, what do you think are the most important challenges to communication?

**Conclusion**

- Thank you for participating. This has been a very useful discussion. I would like to remind you that any comments featuring in this report will be made anonymous
