# Supplementary material for: Collaborative and co-Ordinated action for Medication Safety (COMS): Experience-based co-design of an intervention blueprint to improve general practice and community pharmacy collaboration
Source: PLoS One. 2025 Dec 26;20(12):e0338644. doi: 10.1371/journal.pone.0338644 (PMC12742773; doi:10.1371/journal.pone.0338644)
Supplement: S1 Table — (DOCX) [file pone.0338644.s002.docx]

| **Work system component** | **Subtheme** | **Illustrative quotes** |
| --- | --- | --- |
| **Tools** | **Telephone communication**  **Text/WhatsApp messaging**  **Email communication**  **Instant messaging**  **Google forms**  **Electronic prescribing system**  **Uni-directional communication channels** | *“If I need to make a phone call to a community pharmacy, if it's about patient safety related incident… I can never guarantee whether they are gonna answer the phone, such is the busy nature of the community pharmacy”* GPPT2  *“It was a really helpful conversation but I’ve been in this position now for two and a half years and this was the first time I’ve had contact with this GP surgery. If you phone them, you cannot get through, they just put the phone down on you. It’s difficult.”* CP3  *“…quite a lot of community pharmacies just don’t answer their telephone, so I mean, it’s annoying for me when I’m trying to get hold of them, so I can imagine for a doctor if you’ve tried that a couple of times and nobody’s answered, you’re going to get a bit fed up with phoning pharmacies. So I don’t know what the answer to that is because there are times when you just need to speak to someone at that time. I just think community pharmacy is under such strain at the moment that people just don’t have the resources to do that, which is difficult”.* R1  *“… our community pharmacy contacts can, you know, always just message us and say ‘Oh please can someone give me a ring’... if they need to get hold of us, instead of trying to get through to the surgery.”* GPP2  *“things like WhatsApp groups and things are great. But I don't know how the rest of you are? I don't have a work mobile. I don't particularly want every community pharmacist in the area having my personal details, and when I go home at the end of the night, I'd kind of rather not still be getting messages...so if it's as basic as having almost a system you can log on to or having a handheld device or something that's allocated to you. That would be useful.”* GPP6  *“Obviously different surgeries are different, but I can send an email and just not get a reply”* CP5  *“It is still a slow way of communicating. You know, could take hours before you're getting replies or it might go a day over. It is slow. But at least we have got that trail.”* R2  *“I know certain pharmacies very well and other pharmacies I know less well, and I don't know, is that the e-mail that they’re using?”* GPPT1  *“I would say one of the bigger barriers is technology really, and a lot of community pharmacies don't have more than one computer, or they might have one computer in the dispensary and then one in the consultation room, which isn't convenient to be able to use.”* GPP6  *“So normally they send us a task, a quick task or an instant message, actually, because the patient, normally they go just to the pharmacy next door in the surgery and if it is that, it’s very good because they just send us an instant message saying that this is what has happened.”* GP2  *“We started off by having a WhatsApp group to begin with and then the pharmacies would let us know what stock they had, of antibiotics, and then we started up a Google form. So the pharmacies go on there every day and put in exactly how much quantity they had.”* GPPT3  *“… if there was a prescription error from general practice, that, you know, was picked up and it was actually said ‘We wanna change that’ or… you've gone to cancel that prescription but it's in the community pharmacies possession so they can't cancel it. You have to have to phone them… But that's quite difficult.”* GPPT1  *“if it's only one way and not two-way, it's not really facilitating open communication, is it? It's more like, we, the doctors, are telling you this, rather than like, actually, you're the experts in medicine, can we just have a dialogue in the middle. If our computer systems are not allowing it, then that’s a bit of a barrier.”* GP3  *“That's the only thing that I know that EMIS can do with community pharmacy, is physically, you can do a referral… But when that information comes back, it comes back in a different way… If there was a way that it would integrate, that would be like the best thing.”* GPPT3 |
| **Environment: Socio-organisational** | **Implicit hierarchy**    **Lack of collaborative patient safety analysis and learning**  **Staff continuity**  **Workload/busyness**  **Opening times**  **Access to patient clinical records**    **Variability in approach to communication** | *“And I guess what’s helped community pharmacists working in general practice, I think the GPs have realised that there’s actually some value in…that community pharmacies are quite knowledgeable people, I don’t think they really had much of an idea about what value we could add”* CP1  *“Sometimes the doctors do ring him [the pharmacist] directly if there’s a problem, if it’s a contentious issue, he says one thing and the doctor…overriding the doctor in real terms. And that sometimes has happened. And then the doctor obviously doesn’t like that, gets on and tells him exactly what, you know, needs to be done.”* R1  *“I was thinking beforehand actually of when things go wrong, do we share any root cause analysis with them or is it just you should do a root cause analysis and it's left at that? I think it's more the latter. I think rarely is there any properly shared learning that's on an official system. I think if your relationships are good, you have the confidence to share some constructive feedback both ways. But if that relationship isn't there, then it just sort of gets lost in the ether.”* GP1  *“I saw, like, a few of my GP friends today, so I just asked them, like, oh do you guys have any formal way of getting feedback from the pharmacies about any safety errors, and everyone was like, no, not at all.”* GP3  *“If you are getting a locum pharmacist every day, five days a week… Some of them don't know the system.”* GPPT4  *“I mean, one of the difficulties, I suppose is, you know, communicating to the community pharmacy. Quite often nowadays, this includes pharmacy colleagues that are incredibly stretched and it's very, very busy and they’ve had a really rough few years, but I think it's almost fairly universal now that if I need to make a phone call to a community pharmacy, if it's about patient safety related incident, if it's a prescription that we need to amend, if it's anything like that, I can never guarantee that you're gonna speak to anyone, whether they are gonna answer the phone, such is the busy nature of the community pharmacy.”* GPPT1  *“Sometimes they appreciate how busy it is because they will come across and they’ll have to wait in the queue like everyone else does for whatever they’ve come for. So, they can see we’re not just sat there twiddling our thumbs, the phones are ringing off the hook, there are queues at the desk, and they’re aware that they’ve got to wait and stuff.”* R5  *“Obviously, we’ve sent him a task to issue another medication, for example, but they’re just back-to-back seeing patients. So, a lot of the queries will be done after surgery, where sometimes it might be half-past six till the doctor’s got back to us, and obviously, the chemist closes at six o’clock. So, that kind of thing is what…I mean, is when it is difficult.”* R5  *“If they can see recent consultations as well as clinical letters, discharge letters or things from consultants, that will help them so much to distinguish or find something out before even ringing the surgery.”* GPPT1  *“it's difficult to get a unified system across primary and secondary care, never beyond to social care, community pharmacies. But that would be a golden first step, just as EPS has enabled so much for us to send prescriptions up and down the country and make lives easier for patients.”* GP1  *“It goes back to the classic bit about communication. You know, it irks me all the time that it seems that intelligent people, and I'm not just talking about pharmacists now and pharmacy technicians, I'm talking about all professionals, cannot work out what is the correct form of communication for the type of incident that's in front of them. Maybe we do need expectations of ‘if it's this type of incident, do this’, you know, ‘use this form of communication’.”* GPP5  *“…standardisation of communication between pharmacy and GPs directly for any kind of queries that the GP could resolve. Like, for example, when we were talking about discharges and there’s issues with that, you know, like if there’s a universal standard communication that would be really helpful.”* CP2  *“Everybody's under so much pressure to get workload done and complete, and sometimes it might be that you've got two or three things that you'd like to just run past them and say ‘Right, how do you want to deal with this in the future?’.”* GPPT2 |
| **Environment: physical and external** | **Proximity**  **Co-location**  **Medication shortages** | *“Whereas, [name of GP practice] it was literally right next to the pharmacy. So, that was good, again, I think having that proximity really helps. Because if you've got a minute in-between patient, you kind of literally just bob in and speak to them, so that definitely helps.”* GP3  *“I think because we’ve got a pharmacy attached to the medical centre, they’re open until 11 every night, they’re very good, so most of these sorts of patients are anyway…so the pharmacy they can see all our notes and everything, because they can access all the information so it’s very useful.”* GP2  *“If whatever they wanted is not in stock, then they just bounce back to us, rather than coming back with a list of, like, well this is what is in stock. So, then, we just have to guess, again, what the next thing might be in stock, and send them away again, and sometimes they come back three or four times, and they're furious. And I've always thought, that could just be so simple as, if they had access to EMIS, just a message to say, this is what is in stock, instead of, like, ten phone calls, you know.”* GP3  *“But it was hard for the community pharmacies and for ourselves in GP clinics, especially with Strep A. But yeah, out of stock items is every day. I must get 20 tasks a day from the surgeries saying ‘Somethings out of stock. Need an alternative’.”* GPPT4 |
| **People** | **Familiarity**  **GP pharmacists as boundary spanners**  **Receptionist/prescription clerk role**  **Patient as intermediary**  **GP specific approach to prescription writing/clarity** | *“What else did she do…just generally picking up medication errors. Because she's obviously very approachable, we all knew her very well, there was no issue at all with her telling us that we'd done something wrong, like, it wasn’t going to cause any offence to anyone, because everyone is friends with her.”* GP3  ***Face to face contact***  *“I don't know if it's a personal thing or whether actually it's just that actually most of your communication is non-verbal, actually it's body language. And maybe putting a face to it just gives you that little bit more confidence that you understand the person a bit more and you have common ground a bit more. Maybe it's just that actually face to face contact is a bit more scarce, particularly after COVID.”* GP1  ***Introductions***  *“I just think seeing them and making a bit of effort to perhaps pop into surgeries and introduce yourself makes a big difference, and even if the receptionists like you, that’s helpful.”* CP1  ***Reciprocity***  *“[I] think it’s about trying to work in such a way that there’s benefits to both sides, so how can you help the surgery with something and they help you with something.”* R1  *“What I also find is more and more pharmacists working in primary care, it feels as if the bridge is building slowly, because pharmacists are proving their own abilities in terms of usefulness, knowledge, they can provide within the care settings, and I think they’ve never had the chance, because here community pharmacy are busy in their own worlds, so are GPs in their own world”.* CP3  *“It's better now with the pharmacy team inside the GP practice, because I think the pharmacies do get things sorted a lot quicker. And I think it's because we know what community pharmacies are like.”* GPPT4  *“It’s sometimes easier sharing issues with a practice pharmacist, in my experience. It’s handy to just go, okay, these are the things that have gone wrong. I think the difficulty certainly… is that it’s tricky not to become a sort of negative exercise.”* CP1  *“She [GP practice pharmacist] was like a go-between with the pharmacy, so she knew the pharmacists there, pretty well. And it's like introducing the middle-man, but it's not really, because she had a good relationship both sides.”* GP3  **Gatekeeper role**  *“I don’t think we’ve moved on from the having to call and wait for ages for someone to answer the phone… and then often the receptionist is hesitant to give you any information.”* CP1  *“I think receptionists could be the barrier that you are never able to cross, but I think I’ve seen it and experienced it, especially when I’ve worked in smaller community pharmacies, which is really small community but I think to get passed through the receptionist is your hardest thing. Once you’ve got to the GP, they’re lovely, they really want to work together with you but it’s just unfortunate we cannot just get through.”* R3  ***Education***  *“…our more experienced prescription clerks are very good at their job, they can spot like important…warfarin, for instance…I think they just have a sense…they have enough clinical sort of knowledge after doing it for…of the things that can go wrong, so they're a bit more vigilant for tablets that are particularly important. So experience is a big one because we are essentially delegating tasks with potentially some clinical questions with it to someone doesn't have clinical knowledge.”* GP1  *“I think personally education, educating of GP support staff like receptionists about understanding pharmacists are also clinicians and are only calling the GP for a valid reason and we’re not there to waste their time... So I think just educating the teams on both sides, whether it’s pharmacy support staff and GP receptionists to say if a pharmacist calls or a GP calls to allow that two-way conversation..”* CP4  *“It would be nice if we…because we do have a guy who comes in who’s a pharmacist and I would imagine he would know these things. So if he could sit with us and show these things, you know, it would be…to bridge the gap a bit.”* R2  *“…half the time it [medication safety concerns] comes back from patients, it's like hearsay, which is also not very helpful, and it irritates the patients, if they're the ones having to tell us that we've made a mistake. And they say, oh I went into the pharmacy and they said, this can't possibly be right. So then, you know, they get annoyed with us, because it doesn't seem very professional and it's a bit hard to work out what the pharmacist has actually said, because you're getting a second-hand story.”* GP3  *“…five years ago, I might have seen maybe a piece of paperwork or the pharmacies ring us and say, you know, this person's blood pressure's a little bit high, would you see them today, I'm increasingly having patients being like, I've been to the pharmacy, they asked me to book an appointment. So I think there maybe has been a shift there.”* GP1  *“…where it’s a BD dose maybe, so for instance, phenoxymethylpenicillin, instead of giving it four times, I think it’s just a BD dose but it’s at a higher strength, and just explaining that as per the [name of region] Formulary, so it’s just clearer to the pharmacist that this is okay, so that they don’t again have to query it, that’s all.”* GP2  *“what would be helpful is for a GP if they prescribe some unusual dose, to put please note patient’s renal function is…that’s why I’ve given the lower dose. So EPS2 has enabled much better information-sharing, it’s just that it’s not always used or it is rarely used. So if I was giving feedback to GPs, I would be saying if you’re doing something unusual just put a little note on the script to tell us why because it makes life so much easier.”* CP1 |
